# Therapeutic Implications of PTEN in Non-Small Cell Lung Cancer

**DOI:** 10.3390/pharmaceutics15082090

**Published:** 2023-08-05

**Authors:** Zaid Sirhan, Rawan Alojair, Anita Thyagarajan, Ravi P. Sahu

**Affiliations:** Department of Pharmacology and Toxicology, Boonshoft School of Medicine, Wright State University, Dayton, OH 45435, USA; sirhan.2@wright.edu (Z.S.); alojair.2@wright.edu (R.A.)

**Keywords:** non-small cell lung cancer, PTEN, cancer chemotherapy, targeted therapy

## Abstract

Lung cancer remains one of the major human malignancies affecting both men and women worldwide, with non-small cell lung cancer (NSCLC) being the most prevalent type. Multiple mechanisms have been identified that favor tumor growth as well as impede the efficacy of therapeutic regimens in lung cancer patients. Among tumor suppressor genes that play critical roles in regulating cancer growth, the phosphatase and tensin homolog (PTEN) constitutes one of the important family members implicated in controlling various functional activities of tumor cells, including cell proliferation, apoptosis, angiogenesis, and metastasis. Notably, clinical studies have also documented that lung tumors having an impaired, mutated, or loss of PTEN are associated with low survival or high tumor recurrence rates. To that end, PTEN has been explored as a promising target for anti-cancer agents. Importantly, the ability of PTEN to crosstalk with several signaling pathways provides new approaches to devise effective treatment options for lung cancer treatment. The current review highlights the significance of PTEN and its implications in therapeutic approaches against NSCLC.

## 1. Introduction

Lung cancer remains a major cause of cancer-associated mortality in both men and women in the United States and around the world [1,2,3,4,5]. In addition, lung cancer has been documented to be the number one cause of cancer-related deaths in men and the second cause for women after breast cancer [4,6]. Of two subtypes, non-small cell lung cancer (NSCLC) represents 80–85%, and small cell lung cancer (SCLC) represents 15–20% of lung cancer cases [3,7]. Based on the cell types or site of origin, NSCLC is generally subcategorized into squamous cell carcinoma, adenocarcinoma, and large cell carcinoma [8,9,10]. Among the common risk factors, tobacco smoking has been regarded as one of the main contributing factors to lung carcinogenesis, and others include respiratory diseases such as chronic obstructive pulmonary disease (COPD), occupational exposure to certain heavy metals or radiation, and environmental and genetic factors [11,12,13,14].

While the prognosis of lung cancer remains relatively poor, an initial evaluation can be conducted depending on the history of the patients, laboratory tests, and chest radiography [15,16]. Furthermore, histologic diagnosis is another method that can be implemented if the patient is suspected to have lung cancer. In a significant number of lung cancer patients, the tumor cells metastasize to other organs, largely to lymph nodes, bones, adrenal glands, and the brain [17,18,19,20]. Notably, the tumor type and stages of the cancer are used to determine the prognosis and treatment options [21,22]. In early-stage NSCLC, lobectomy is a preferred choice of treatment [21,23,24,25]. However, for the advanced-stage and unresectable NSCLC, radiotherapy, chemotherapy, and a combination of chemotherapeutic agents or targeted therapy have been explored [26,27,28].

Of importance, targeted therapy is specifically designed to eradicate tumor cells that harbor activating mutations in any particular gene of a signaling cascade and is currently being implemented to treat advanced-stage or metastatic lung cancer patients [29,30]. For example, erlotinib and gefitinib are tyrosine kinase inhibitors (TKIs) that target tumor cells with activating mutations in the epidermal growth factor receptor (EGFR) gene [31,32,33]. While such approaches are becoming the standard of care for lung cancer treatment, their effectiveness is often compromised via counteracting mechanisms, indicating the need for other alternative approaches for lung cancer treatment [34,35].

### Biology and History of PTEN

The oncogene-driven paradigm of cancer predominated from the 1970s through the 1980s, and it was proposed that the major driver of transformation is the overexpression or aberrant activation of certain genes [36,37]. However, Henry Harris and his peers’ work demonstrates that the fusion of a cancer cell with a normal cell might reduce tumorigenicity and provide the foundation for the tumor suppressor period. This groundbreaking study proposed that there is an “unknown” factor/mediator that is contributed by normal cells to the hybrid cells that decrease the tumor cells’ highly malignant nature [38]. The identification of Harris’ “unknown” factor/mediator was the next significant obstacle and provided the most likely reason for the prolonged latency for the formation of a tumor suppressor-driven model of cancer [36]. Unknown at the time, a breakthrough soon followed in the form of an epidemiological study of retinoblastoma patients, where Alfred Knudson created a statistical model that proposed that two different genes must experience mutations for retinoblastoma tumors to develop [39]. The 13q14 locus was subsequently found to often be deleted in these tumors, which was quickly followed by the identification of the retinoblastoma (Rb) gene [40,41,42,43].

Importantly, Knudson’s two-hit theory was explicitly supported by the finding that both Rb alleles were lost [39]. According to this theory, cancer is caused by the deletion or inactivation of the other Rb allele, not by a mutation that leads to the loss of one Rb allele [41,42,43]. Due to the identification of tumor suppressor genes that were crucial in the development of numerous cancers, the tumor suppressor period advanced significantly from the middle of the 1980s until well into the 1990s. The capacity to pinpoint the location of allelic loss to particular chromosomal sites signaled this. This method was used to identify potential tumor suppressors, which included the previously discovered Rb gene on chromosome 13q14. Similar to this, cytogenetic and molecular investigations in the 1980s showed that chromosome 10q23 was frequently partially or completely lost in prostate and brain tumors, indicating the presence of yet another significant tumor suppressor gene on chromosome 10 [44,45,46,47,48,49]. The identity of a frequently lost tumor suppressor on human chromosome 10q23 was not made public until 1997, or about 25 years ago now. Two independent researchers, one group led by Ramon Parsons and the other by Peter Steck, cloned and characterized the phosphatase and tensin homolog (PTEN), which is mutated in multiple advanced malignancies (MMAC), respectively [44,50,51,52,53]. Although Steck et al. studies suggested that PTEN may play a role in hereditary disease, Charis Eng and Ramon Parson’s team were the first to explicitly acknowledge PTEN as being targeted by germline mutations in individuals with the cancer predisposition syndrome known as Cowden disease [36,40,54].

**Figure 1:**
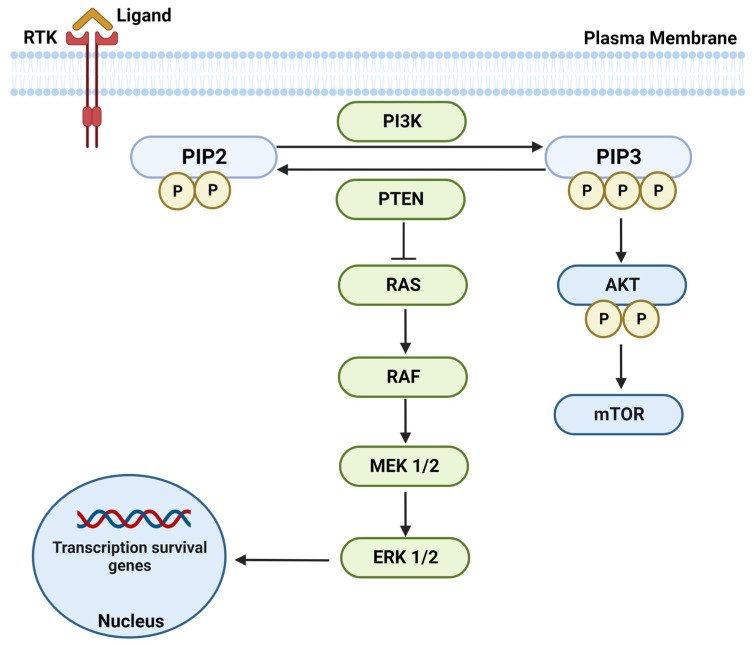
The schematic representation of PTEN’s function and associated signaling pathways’ controlling gene transcription and regulating cell survival and proliferation. The binding of the growth factor ligands to the receptor tyrosine kinase (RTK) activates PI3K, which phosphorylates PIP2 to PIP3, resulting in the activation of the downstream AKT-mTOR pathway. PTEN antagonizes PI3K activity and inhibits the downstream RAS-RAF-MEK-ERK signaling, leading to the inhibition of the transcription of survival genes.

As detailed above, among various cellular targets, the phosphatase and tensin homolog (PTEN) is one of the important family members of tumor-suppressive genes, which has been extensively studied in preclinical and clinical settings of lung cancer [55,56,57]. PTEN plays an essential role in regulating various cellular activities, including cell migration, cell cycle arrest, apoptosis, angiogenesis, and metastasis [56,57,58]. The best-characterized tumor-suppressive role of PTEN is the lipid phosphatase activity that antagonizes phosphatidylinositol 3-kinase (PI3K) signaling. Also, PTEN inhibits the PI3K/mammalian target of the rapamycin (mTOR)/AKT signaling pathway, which governs cell growth, survival, and migration by dephosphorylating phosphatidylinositol 3,4,5 triphosphate (PIP3) to phosphatidylinositol 4,5 bisphosphate (PIP2). This results in the inhibition of functional cellular activities, including cell proliferation, survival, and migration [56,58,59]. In addition to negatively regulating the PI3K/mTOR/AKT pathway, PTEN has been shown to target downstream mitogen-activated protein kinases (MAPK) RAS-RAF-MEK-ERK signaling, leading to the inhibition of the transcription of survival genes and playing critical roles in DNA repair and preserving chromosomal integrity [55,60]. The schematic representation of PTEN’s function and its associated pathways is shown in Figure 1.

However, an impaired and/or loss of the functional activity of PTEN is a major determinant in impacting tumor development across all cancer types [61,62]. To that end, multiple mechanisms have been proposed for PTEN inactivation, such as the decreased expression of its protein levels, germline and somatic mutations, promoter hypermethylation, or phosphorylation, leading to a loss of its phosphatase activity [50,63]. Furthermore, several modifications have also been shown to cause a loss/deletion or mutations in the PTEN gene, which could lead to alterations, including in protein–protein interactions, transcriptional silencing, post-translational modifications, and inherited germline mutations [64]. Also, the homozygous loss of PTEN has been shown to partially uncouple mutant EGFR from its downstream pathway and activate EGFR, leading to the segregation of EGFR-dependent and EGFR-independent cells, which results in the development of therapeutic drug (e.g., erlotinib) resistance [56,65]. Importantly, as the mutant/loss of PTEN limits the efficacy of standard-of-care therapies, including radiotherapy, deregulation in the ataxia telangiectasia mutated kinase (ATM) that acts as a prime sensor of double-strand DNA breaks, has been documented in PTEN mutant tumors [66]. Notably, the inhibition of ATM has been found to sensitize PTEN-deficient/mutant human and murine NSCLC cell lines and organotypic lung tumor slice cultures to radiotherapy [66].

On the other hand, several strategies have been explored to induce PTEN expression/ activity with the overall goal of regulating the signaling pathways involved in cancer growth and resistance to therapeutic agents. To that end, the treatment with naturally occurring phytochemicals such as curcumin, indol-3-carbinol, genistein, resveratrol, and sulforaphane has been found to induce PTEN expression or activity [67,68,69,70,71,72,73]. Furthermore, the gene therapy-based branch-PCR-assembled PTEN gene nanovector (NP-PTEN) approach has been explored to overexpress PTEN protein expression and restore its functions via inhibiting the activation of the PI3K/AKT/mTOR pathway in NSCLC cells [74]. Importantly, these NP-PTEN-mediated effects have been found to inhibit in vitro cell proliferation and induce apoptosis, as well as suppress the in vivo growth of tumor xenografts, indicating the anti-cancer efficacy and therapeutic potential of this gene therapy approach for NSCLC [74]. The current review highlights recent updates on PTEN and its regulated signaling pathways, which uncovers the gaps in the mechanistic insights for its role in NSCLC.

## 2. Implications of PTEN in NSCLC

### 2.1. In Vitro and In Vivo Studies

Several studies have used in vitro culture systems and mouse models to define the functional significance of PTEN in NSCLC. The summaries of the in vitro studies that included the overexpression of PTEN in NSCLC cell lines and in vivo studies are given in Table 1. In one study, Lu and colleagues investigated the crosstalk of PTEN with hTERT and the PI3K/AKT pathway, using the lung adenocarcinoma A549 cell line overexpressing wild-type or mutant PTEN, or the PTEN siRNA approach [75]. The result showed that PTEN plays a vital role in suppressing cell proliferation via inducing cell cycle arrest and apoptosis. In comparison to the control group, it was found that the mRNA and protein levels of hTERT were lower in the A549 cell line transfected with wild-type PTEN. Also, in A549 cells transfected with PTEN-siRNA and hTERT mRNA, protein levels were significantly higher than in the control group. These findings show that PTEN inhibits hTERT expression in NSCLC cells. The PTEN mRNA and protein levels increased when the A549 cells were transfected with the phosphatase-dead PTEN mutant, but there was no change in hTERT expression levels, indicating that the phosphatase-dead PTEN mutant is nonfunctional and that only wild-type PTEN suppresses hTERT expression. Mechanistically, it has been observed that PTEN suppressed cell proliferation by inhibiting the PI3K/AKT/hTERT pathway. Overall, these findings indicated that the downregulation of the PI3K/AKT/hTERT pathway is one of the PTEN mechanisms that acts as a tumor suppressor in lung cancer [75].

Moreover, the PTEN expression in lung cancer cells is regulated by deubiquitylase Ataxin-3 [76]. For the identification of deubiquitylates (DUBs), Sacco and colleagues used an unbiased siRNA screening approach and found that PTEN expression is affected by the PI3K pathway. The members of the DUBs family consist mainly of ATXN3, ATXN3L, and JOSD1, as well as other factors, like PTENP1 and PTEN transcripts, and its RNA levels increased markedly as the reduction in each of the DUBs occurred [76]. The degradation of the PTEN protein did not affect the DUBs. Importantly, the PTEN induction observed in response to the ATXN3 siRNA was found to sufficiently downregulate AKT phosphorylation and, hence, the PI3K signaling. Also, histone deacetylase inhibitors (HDACi) have been suggested as potential mediators of PTEN transcriptional reactivation in NSCLC. The authors found that although PTEN exhibited a very limited response to the broad-spectrum HDACi, vorinostat (SAHA), in A549 cells, its combination with ATXN3 depletion enhanced PTEN induction in an additive manner. Similarly, these interventions additively decreased the cell viability. These findings indicated that ATXN3 acts as an autonomous target for therapeutic intervention for lung cancer, which is associated with an epigenetic downregulation of PTEN [76].

In addition, pemetrexed treatment for lung cancer cells has also been documented to inhibit the PI3K pathway. In a study, Li and colleagues demonstrated that PTEN overexpression can increase the efficacy of pemetrexed in the A549 NSCLC cell line [77]. Importantly, the combination of pemetrexed with PTEN overexpression inhibited the AKT signaling pathway; however, the mTOR signaling pathway was found to be activated, which resulted in the induction of apoptosis-associated genes, including p53, Bcl2, and BAX. In addition, the results suggested that treatment with pemetrexed combined with PTEN overexpression can inhibit the aerobic oxidation of glucose, which decreases the energy supply of cancer cells, leading to increased apoptosis. Overall, the data indicated that pemetrexed treatment inhibits the proliferation of NSCLC cells via targeting the PI3K/AKT/mTOR signaling pathway and carbohydrate metabolism, and thus, it represents a novel therapeutic strategy for the treatment of NSCLC [77].

Importantly, Noro and colleagues determined how PTEN inactivation can affect tumor progression and drug resistance in lung cancer [78]. By using a panel of lung cancer cell lines, the authors examined the levels of PTEN expression at both the mRNA and protein levels and their genetic and epigenetic status. Low expression of the PTEN protein was documented in six cell lines out of the twenty-five cell lines tested. Out of the six cell lines having low PTEN expression, the genomic analysis of the QG56 and N23 cell lines showed homozygous deletions of the PTEN gene. By using a methylation-specific PCR, the authors showed that the PC10 and PC14 cell lines have hyper-methylation of the PTEN gene promoter. After treatment with the demethylating agent, 5-aza-2′deoxycytidine (5-AZA), and the histone deacetylase (HDAC) inhibitor, trichostatin A (TSA), the authors observed that the gefitinib and TSA combination induced significant growth inhibition in two gefitinib-resistant cell lines. Overall, these findings suggested that the combination of gefitinib with the demethylating agent, 5-AZA, and TSA could be beneficial for treating NSCLC [78].

As PTEN deletion has been shown to occur within the tumor cells residing in the airway basal cells, Malkoski and colleagues performed a study using a mouse model to examine if the loss of PTEN in airway basal cells can initiate tumor formation or increase squamous cell carcinoma formation [80]. The authors have previously established that targeting Kras^G12D^ activation and transforming growth factor β receptor type II (TGFβRII) deletion to airway basal cells via a keratin promoter resulted in the development of both adenocarcinoma and squamous cell carcinoma in the lungs. The authors found that when PTEN deletion occurs in targeting basal cells, it can initiate both lung adenocarcinoma and squamous cell carcinoma formation. Although PTEN deletion is a weaker tumor initiator than Kras^G12D^, with low tumor multiplicity and long latency, tumors initiated by PTEN deletion were larger and more malignant than Kras^G12D^-initiated tumors. When genetic modifications were focused on a particular cell, the fact that PTEN loss did not enhance lung SCC development in comparison to Kras^G12D^ activation implies that the initiating genetic event does not determine tumor histology. These findings also indicated that conducting airway basal cells can give rise to a variety of NSCLC tumors [80].

Along similar lines, Yu and colleagues determined the mechanisms and functional significance of the abnormal expression of PTEN using various in vitro and in vivo models of NSCLC [79]. The authors demonstrated that PTEN knockdown induced increased proliferation of the H1975, A549, HCC827, and H1650 cell lines, including a colony formation assay, and resulted in the enhanced growth of subcutaneously implanted tumor xenografts. The overexpression of PTEN rescued the loss of the PTEN phenotypes, while suppression of the PTEN expression via the shRNA approach was found to increase the number of metastatic tumors. These studies also revealed that the integrin αVβ6 signaling pathway was affected by PTEN [79]. As lung cancers have higher levels of integrin αVβ6 expression, this study found that the overexpression of PTEN led to the suppression of cancer progression by inhibiting the integrin αVβ6. Importantly, the data demonstrated that PTEN has the potential to be explored as a therapeutic target because of its tumor-suppressive activity [79].

Notably, protein ubiquitination plays a dynamic role in modulating protein stability, including in PTEN, and it requires ubiquitin-specific proteases (USPs) or deubiquitinases (Dubs) to hydrolyze the ubiquitin molecules from their substrates. In a study, He and colleagues determined the effect and mechanism of USP10 as a tumor suppressor Dub of PTEN [81]. The authors previously established that an E3 ubiquitin ligase, tripartite motif-containing 25 (TRIM25), induces the K63-linked polyubiquitination of PTEN and inhibits its phosphatase activity [85], which provided the rationale to explore Dubs in this effect. Using immunoprecipitation/immunoblotting assays, the authors found USP10 to be the most potent Dub in suppressing PTEN K63-linked polyubiquitination mediated by TRIM25. Further studies confirmed that USP10 interrupted the interaction between PTEN and TRIM25 yet did not affect the mRNA or protein stability of PTEN [81]. Importantly, USP10 expression was downregulated/low in the NSCLC cell lines and in the primary NSCLC tissues compared to the normal human bronchial epithelial cells and matched normal tissues. Mechanistic studies demonstrated that USP10 suppresses the activation of the AKT/mTOR pathway via decreasing the K63-linked polyubiquitination of PTEN, which resulted in the inhibition of the cell viability, proliferation, and migration of the A549 and H1299 NSCLC cell lines. Overall, these studies demonstrated that USP10 targets the PTEN/AKT/mTOR signaling pathway in NSCLC by preventing K63-linked polyubiquitination of PTEN and, thus, acts as a tumor suppressor.

Of importance, the role and mechanisms of the RNA-binding motif protein 10 (RBM10), an alternative splicing regulator that plays an important role in regulating proliferation and apoptosis, was determined in NSCLC [82]. An analysis of NSCLC tissues from patients and the NSCLC A549 and H1299 cell lines revealed lower expression of RBM10 as compared to paired paracancerous tissues and the human bronchial epithelial cell line (BEAS-2B). The overexpression of RBM10 was suppressed, and its silencing enhanced invasion and migration, as well as EMT-related protein expression, indicating its tumor suppressor activity. Mechanistically, these effects were found to be mediated via RBM10-induced PTEN expression and inhibition of the activation of the PI3K/AKT/mTOR pathway [82]. Furthermore, clip-seq analysis demonstrated that RBM10 interacts with 353 long non-coding RNAs (lncRNAs), and among these, it binds to the nuclear enriched abundant transcript 1 (Neat1) with higher affinity and also regulates its splicing variants. Importantly, RBM10 overexpression was reduced, and its silencing increased Neat1 expression, indicating that RBM10 regulates the alternative splicing (AS) of Neat1 [82]. Further studies confirmed that induced RBM10 increased PTEN expression and decreased PI3K/AKT/mTOR activation and was mediated via Neat1 splicing variants. These studies indicated that RBM1 targets the PTEN/PI3K/AKT/mTOR/Neat1 axis to regulate the growth of NSCLC cells.

Along similar lines, studies by Cai and colleagues demonstrated that casein kinase 1 alpha 1 (CK1α), a negative regulator of the Wnt pathway, induces PTEN stabilization and activity by preventing PTEN and NEDD4-1 binding and inhibiting PTEN polyubiquitination and phosphorylation [83]. This effect resulted in the inhibition of AKT activity and the upregulation of the FOXO3a-induced transcription of the E1 enzyme, Atg7, which plays a crucial role in inducing autophagy. Mechanistic studies demonstrated that the CK1α/PTEN/FOXO3a/Atg7 axis inhibits the growth of A549 NSCLC cells and A549 lung tumor xenografts via inducing autophagy and that blocking CK1α-induced autophagy mediates oncogenic HRas^V12^. Importantly, high expression of CK1α, PTEN, and Atg7 in human NSCLC tissues was found to be associated with increased overall survival and vice versa. Overall, these findings indicated that PTEN interacts with CK1α, which maintains PTEN stability and activity to induce autophagy in NSCLC.

In one study, He and colleagues determined the role and mechanism of protein tyrosine phosphatase interacting protein 51 (PTPIP51) as a tumor suppressor and its interaction with PTEN in NSCLC [84]. Using an integrated bioinformatics approach and NSCLC tissue samples and their matched normal tissue samples, the authors demonstrated that PTPIP51 expression is downregulated in NSCLC, and its low expression was associated with poor patient survival [84]. An analysis of PTPIP51 in NSCLC patients harboring EGFR mutations and treated with a targeted kinase inhibitor showed its significantly increased expression, which was associated with an overall improved objective response rate (ORR). To determine the functional significance of PTPIP51 in NSCLC, the authors overexpressed PTPIP51 in the PC9 and A549 cell lines and found that it impairs in vitro cell proliferation, induces apoptosis, and enhances the sensitivity of gefitinib. The in vivo studies with a patient-derived xenograft (PCX) mouse model demonstrated that the intratumoral injection of Ad-PTPIP51 inhibits tumor growth and also increases the sensitivity of gefitinib. Mechanistic studies demonstrated that PTPIP51 not only inhibits EGFR activation but the total EGFR protein levels via promoting its ubiquitylation and subsequent degradation. As PTEN plays a crucial role in EGFR degradation, further studies showed that PTPIP51 directly interacts with PTEN and induces its activation via the CK2 protein kinase and that PTPIP51-PTEN-CK2 complex promotes EGFR degradation [84]. Overall, these findings demonstrated that PTPIP51 acts as a tumor suppressor in NSCLC, and its effects were mediated via PTEN activation and EGFR degradation.

### 2.2. Clinical Studies

Several studies have investigated the impacts of somatic mutations in PTEN in NSCLC patients. The summaries of the clinical studies are given in Table 2. In one study, Jin and colleagues determined the correlation between PTEN mutations and EGFR, KRAS, and TP53 mutations in tumor samples collected from a large cohort of NSCLC patients with or without smoking status [86]. The genetic analysis of the various exons of these genes showed that the prevalence of PTEN mutations was 4.5% (eight out of one hundred and seventy-six cases) of patients having a history of smoking. Importantly, 50% of the NSCLC patients with PTEN mutations also had P53 gene mutations. However, EGFR mutations were found to be infrequent (one out of eight cases) among the NSCLC patients harboring PTEN mutations, and none of these patients had KRAS gene mutations. These findings indicated that a subset of PTEN mutation-positive patients exhibit a positive correlation with the P53 and EGFR genes [86].

In addition, Sos and colleagues used a vast collection of genomically defined NSCLC cell lines to identify genomic traits that distinguish EGFR-dependent from EGFR-independent EGFR-mutant lung tumor cells. The authors used a combination of computational, biochemical, and cellular methods to discover new mechanisms that uncouple EGFR-dependent tumors from their downstream signaling. The computational genomics analyses found PTEN homozygous deletion to be a candidate for EGFR inhibitor resistance. The functional studies revealed that PTEN loss causes a considerable decrease in apoptosis sensitivity in EGFR-mutant cells by activating AKT and EGFR, and it was hypothesized that ERK activation in PTEN-deficient cells leads to the increased transcription of EGFR ligands, such as amphiregulin [87].

In another study, Yoo and colleagues evaluated whether surgically resected primary NSCLC and the loss of PTEN expression correlates with clinicopathological parameters or is related to EGFR gene status in NSCLC [88]. The authors analyzed 288 samples for PTEN expression and its correlation with the clinicopathological parameters. It was found that the loss of PTEN expression was associated with 42.4% of the samples, and of those, 28.6% were adenocarcinoma, 66.7% were squamous cell carcinoma, and 38.1% were of an NSCLC subtype. The loss of PTEN expression was significantly associated with smoking, male gender, larger tumor size, and a high pathological stag, while the loss of PTEN expression, age, performance status, and pleural invasion showed no correlation. The survival analysis showed that the progression-free survival was shorter in the group that had a loss of PTEN expression compared to the PTEN intact group. In addition, it was found that PTEN expression was not associated with EGFR status, such as the EGFR copy number or mutation [88].

Of importance, Cumberbatch and colleagues found that clinical SCC specimens had a significantly increased expression of PI3Kβ and reduced/loss of PTEN expression compared to adenocarcinoma specimens [89]. Detailed correlative analyses of individual patient samples revealed a significantly greater proportion of SCC with higher PI3Kβ and lower PTEN expression. Overall, the authors identified, for the first time, a subset of NSCLC, with the more prevalent SCC having an elevated expression of PI3Kβ, which was accompanied by a reduction or loss of PTEN, for whom selective PI3Kβ inhibitors may be predicted to achieve greater clinical benefit [89].

Along similar lines, Panagiotou and colleagues conducted a study to quantitatively analyze the PTEN protein expression in NSCLC. A sample of 61 NSCLC cases was used in the study; about 80% of the cases were males, while 20% of the cases were females. A total of 57% of patients had adenocarcinoma, and 42% had squamous cell carcinoma. Digital image analysis was conducted to analyze the PTEN protein quantitively, and it was found that patients who developed a progressive loss of PTEN expression appeared to have a higher rate of metastases than those with PTEN overexpression. Therefore, in NSCLC, PTEN deregulation was associated with an aggressive phenotype, and this is an important factor in determining a tailored targeted therapy regimen, especially when combined with HER2/PI3K inhibitors [90].

While there are therapeutic potentials to modulate PTEN, it still is an undruggable target. Thus, PI3K/AKT/mTOR inhibitors have been used in clinical settings to treat human malignancies, including lung cancer. A list of ongoing and recently completed clinical trials of PI3K/AKT/mTOR inhibitors is given in Table 3. Notably, some of the clinical trials also used other pathway inhibitors, such as the MEK inhibitor in combination with PI3K/AKT/mTOR inhibitors.

## 3. Conclusions and Future Perspective

Several studies have confirmed the potential implications of PTEN in NSCLC.While the primary mechanism of its tumor suppressor activity is mediated via its ability to dephosphorylate PIP3 into PIP2, resulting in antagonizing PI3K/AKT signaling and inducing tumor cell death, the detailed mechanisms (i.e., downstream pathways and crosstalk with other signaling cascades) are yet to be fully determined. Given that a reduced or loss of tumoral PTEN expression has been associated with the overall low survival of NSCLC patients, approaches to overcome this effect would aid in the ongoing challenges for the treatment of NSCLC patients. Of significance, given the importance of predictive biomarkers in devising promising strategies to decrease the risk and/or improve the overall therapeutic outcomes in NSCLC patients, several studies have supported the applicability of the PTEN status to be explored as a prognostic marker against various malignancies, including NSCLC.

## Figures and Tables

**Table 1:** Summary of the in vitro and in vivo studies defining the role and mechanisms of PTEN in NSCLC.

Cell Lines	Therapies	Target (s)	Findings	Refs.
A549 cells	-	PI3K/AKT/hTERT signaling pathway	PTEN downregulates PI3K/AKT/hTERT pathway to suppress the growth of A549 cells.	[75]
A549 cells	-	PI3K/AKT signaling pathway	ATXN3 provides an autonomous, complementary therapeutic target in cancers with epigenetic downregulation of PTEN.	[76]
A549 cells	Pemetrexed	PI3K/AKT/mTOR signaling pathway	Pemetrexed inhibits the proliferation of lung cancer cells via inhibiting the PI3K/AKT/mTOR pathway and through carbohydrate metabolism.	[77]
Twelve adenocarcinoma cell lines (ABC-1, A549, PC3, PC7, RERF-LCMS, RERF- LCKJ, VMRC-LCD, RERF-LCOK, PC14, PC9, PC9/f9, and PC9/f14). Eight squamous cell carcinoma cell lines (QG56, EBC-1, LK-2, LC-1/sq, PC1, RERF-LCAI, PC10, and SQ5). Five small cell carcinoma cell lines (NCI-H69, SBC3, NCI-N231, and Lu135).	5-AZA, HDAC inhibitor, TSA, and gefitinib	PI3K/AKT signaling pathway	The combination of gefitinib with 5-AZA and TSA exerts synergistic effects in the inhibition of cell survival via targeting the PI3K/AKT pathway and, thus, could be explored for treating lung cancer.	[78]
H1975, A549, HCC827, and H1650 cell lines and female BALB/c nu/nu mouse model.	-	The integrin αVβ6 signaling pathway	Overexpression of PTEN resulted in the suppression of cancer progression by the inhibition of integrin αVβ6.	[79]
Transgenic mouse harboring TGFβRII conditional deletion allele, PTEN conditional deletion allele, lox-stop-lox-(LSL) KrasG12D knock-in allele, K5CrePR, and K14CrePR1 transgenes.	-	-	PTEN deletion in airway basal cells can initiate both lung adenocarcinoma and squamous cell carcinoma formation, and these tumors were relatively larger and highly malignant compared to the tumors initiated via Kras^G12D^ mutation.	[80]
A549 and H1299 cell lines and NSCLC tissues.	-	AKT/mTOT	USP10 inhibits NSCLC proliferation by preventing PTEN from K63-linked polyubiquitination and inhibiting the activation of AKT/mTOR pathway.	[81]
A549 and H1299 NSCLC cell lines and NSCLC tissues.	-	PTEN/PI3K/AKT/ mTOR/Neat1	RBM10 targets PTEN/PI3K/AKT/mTOR/Neat1 axis to regulate the growth of NSCLC cells.	[82]
A549 cells, xenograft model, and NSCLC tissues.	-	CK1α/PTEN/FOXO3a/Atg7	CK1α/PTEN/FOXO3a/Atg7 axis inhibits the growth of A549 NSCLC cells and A549 lung tumor xenografts via inducing autophagy.	[83]
PC9 and A549 NSCLC cell lines, PDX mouse model, and NSCLC tissues.	Gefitinib	EGFR	PTPIP51 inhibits NSCLC proliferation, induces apoptosis, decreases tumor growth, and increases gefitinib sensitivity via PTEN activation and EGFR degradation.	[84]

**Table 2:** Summary of the clinical studies defining the role/mechanisms of PTEN in NSCLC.

Study Design	Therapy	Target (s)	Findings	Refs.
To define somatic mutations in PTEN in small number of NSCLC cases.	-	-	A subset of PTEN mutation-positive patients exhibited a positive correlation with p53 and EGFR genes, and none of these patients had the KRAS gene mutation.	[86]
To determine the role of homozygous deletion of PTEN as a candidate for EGFR inhibitor resistance.	Erlotinib	AKT signaling pathway	The results were centrally related to the co-occurrence of PTEN loss and EFGR mutations. In EGFR-mutant NSCLC, PTEN loss acts as a mechanism of initiation or acquired resistance to erlotinib-induced apoptosis.	[87]
To analyze PTEN expression and the correlation between PTEN expression and clinicopathological parameters in NSCLC.	-	-	42.4% (122/288) of NSCLC patients had a loss of PTEN expression. Squamous cell carcinoma, male gender, and smoking exhibited a correlation with the loss of PTEN expression.	[88]
To identify a subset of NSCLC based on PI3Kβ and PTEN expression.	-	PI3Kβ pathway	In NSCLC patients, SCC was more prevalent with elevated expression of PI3Kβ accompanied by a reduction/loss of PTEN, for whom selective PI3Kβ inhibitors may be predicted to achieve more significant clinical benefit.	[89]
To analyze PTEN expression in NSCLC.	-	-	In NSCLC, PTEN deregulation is associated with an aggressive phenotype, and this is an essential factor in determining tailored targeted therapy regimens, especially when combined with HER2/PI3K inhibitors.	[90]

**Table 3:** Ongoing and recently completed clinical trials of PI3K/AKT/mTOR inhibitors.

Study Title	Study Phase/ Status	Conditions	Intervention	Primary Outcome Measures	NCT Number	Refs.
PI3K inhibitor BKM120, carboplatin, and pemetrexed disodium in treating patients with stage IV non-small cell lung cancer.	Phase I (Completed)	Recurrent NSCLC, Stage IV NSCLC	PI3K inhibitor BKM120, pemetrexed disodium, carboplatin	Maximum tolerated dose (MTD), defined as the highest dose in which fewer than 33% of study participants experience a dose limiting toxicity (DLT), toxicity profile of PI3K inhibitor BKM120.	NCT01723800	[91]
Palliative thoracic radiotherapy, plus BKM120.	Phase I (Completed)	NSCLC	BKM120	Dose escalation analysis: number of DLTs observed in evaluable patients; MTD was defined as the highest dose at which no more than 1 of 6 evaluable patients or 0 of 3 evaluable patients experience DLT.	NCT02128724	[92]
A trial of gefitinib in combination with BKM120 in patients with advanced non-small cell lung cancer, with enrichment for patients whose tumors harbor molecular alterations of PI3K pathway and are known to overexpress EGFR.	Phase I (Completed)	NSCLC, solid tumors	Gefitinib and BKM120	Recommended Phase 2 dose for gefitinib and BKM120 combination therapy.	NCT01570296	[93]
Trial of MEK inhibitor and PI3K/mTOR inhibitor in subjects with locally advanced or metastatic solid tumors.	Phase I (Completed)	Locally advanced solid tumor, metastatic solid tumor, breast cancer, NSCLC, melanoma, colorectal cancer	MSC1936369B (pimasertib), SAR245409 (PI3K), and mTOR inhibitor.	Number of subjects with DLT.	NCT01390818	[94]
A study of the safety and pharmacology of PI3-kinase inhibitor, GDC-0941, in combination with either paclitaxel and carboplatin (with or without bevacizumab) or pemetrexed, cisplatin, and bevacizumab in patients with advanced NSCLC.	Phase I (Completed)	Non-squamous NSCLC	GDC-0941, bevacizumab, carboplatin, cisplatin, paclitaxel, pemetrexed	Number of participants with DLTs; percentage of participants with adverse events (AEs).	NCT00974584	[95]
Study of BGB-10188 as monotherapy and in combination with zanubrutinib and tislelizumab.	Phase I, Phase II (Ongoing)	Chronic lymphocytic leukemia, small lymphocytic lymphoma, follicular lymphoma, marginal zone lymphoma, mantle cell lymphoma, diffuse large B-cell lymphoma, advanced solid tumor, NSCLC, small cell lung cancer, metastatic melanoma	BGB-10188, Zanubrutinib, tislelizumab	The recommended Phase 2 dose (RP2D) of BGB-10188 monotherapy, RP2D of BGB-10188 in combination with Zanubrutinib, RP2D of BGB-10188 in combination with tislelizumab, overall response rate (ORR), number of participants experiencing treatment-emergent adverse events (TEAEs), number of participants experiencing severe adverse events (SAEs), number of participants experiencing AEs leading to discontinuation.	NCT04282018	[96]
A study to evaluate the safety and tolerability of TOS-358 in adults with select solid tumors.	Phase I (Ongoing)	Colorectal cancer, gastric cancer, HER2-negative breast cancer, NSCLC, squamous cell carcinoma of head and neck, urothelial carcinoma, cervical cancer, ovarian cancer, endometrial cancer	TOS-358	Determine the rate of DLTs, incidence, and severity of AEs, and specific laboratory abnormalities.	NCT05683418	[97]
TQ-B3525 tablets combined with osimertinib mesylate tablets in the treatment of advanced NSCLC.	Phase I, Phase II (Ongoing)	NSCLC	TQ-B3525 tablets, osimertinib mesylate tablets	DLT, baseline time to increase chemotherapy drug dose to toxicity intolerance, objective response rate (ORR).	NCT05284994	[98]
Pembrolizumab + idelalisib for lung cancer study.	Phase I, Phase II (Unknown)	NSCLC, metastasis, recurrence	Pembrolizumab, idelalisib	Number of participants with DLT.	NCT03257722	[99]
MK2206 and erlotinib hydrochloride in treating patients with advanced NSCLC who have progressed after previous response to erlotinib hydrochloride therapy.	Phase II (Completed)	Adenosquamous lung carcinoma, bronchioloalveolar carcinoma, large cell lung carcinoma, lung adenocarcinoma, recurrent NSCLC, squamous cell lung carcinoma	Akt inhibitor MK2206, erlotinib hydrochloride	Disease-control rate, response.	NCT01294306	[100]
Study to assess safety, pharmacokinetics, and efficacy of oral CC-223 for patients with advanced solid tumors, non-Hodgkin lymphoma, or multiple myeloma.	Phase I, Phase II (Completed)	Multiple myeloma, diffuse large B-Cell lymphoma, glioblastoma multiforme, hepatocellular carcinoma, NSCLC, neuroendocrine tumors of non-pancreatic origin, hormone receptor-positive breast cancer	CC-223	Number of participants with DLT, maximum observed plasma concentration (Cmax) of CC-223, time to maximum concentration (Tmax) of CC-223, apparent volume of distribution (Vz/F) of CC-223.	NCT01177397	[101]
Study assessing safety, pharmacokinetics, and efficacy of CC-223 with either erlotinib or oral azacitidine in advanced NSCLC.	Phase I (Completed)	NSCLC	CC-223, erlotinib, CC-223, oral azacitidine	Adverse events, number of participants with adverse events, MTD, PK-Cmax, Pk-Maximum observed concentration in plasma (Cmax), PK-AUC, PK-Tmax.	NCT01545947	[102]
Combination of RAD001 with carboplatin, paclitaxel, and bevacizumab in NSCLC patients.	Phase I (Completed)	NSCLC	RAD001	Establish the feasible doses/regimens of RAD001 in combination with chemotherapy. Primary endpoint is the end-of-cycle DLT rate every 3 months or once critical DLT occurs.	NCT00457119	[103]
Combination of RAD001 and erlotinib in patients with advanced NSCLC previously treated only with chemotherapy.	Phase I (Completed)	NSCLC	RAD001, erlotinib	Dose limiting toxicities (DLTs) and PK drug–drug interaction (DDI). Phase 2: Tumor response assessed by CT scans.	NCT00456833	[104]
A Phase I study of SUNITINIB and rapamycin in advanced NSCLC.	Phase I (Completed)	NSCLC	Sunitinib and rapamycin	To define the optimal dose of sunitinib when given in combination with rapamycin 2mg daily, determine the dose limiting toxicity of sunitinib and rapamycin.	NCT00555256	[105]
Study of everolimus, pemetrexed, carboplatin, and bevacizumab to treat stage IV lung cancer	Phase I (Completed)	NSCLC	Everolimus, pemetrexed, carboplatin, bevacizumab	Define the MTD and RP2TD of the combination of everolimus with pemetrexed, carboplatin, and bevacizumab in patients with Stage IV non-squamous NSCLC.	NCT01700400	[106]
Gefitinib and everolimus in treating patients with Stage IIIB, Stage IV, or recurrent NSCLC.	Phase I (Completed)	NSCLC	Everolimus, gefitinib	Overall objective response, efficacy of the combination of daily oral gefitinib and daily oral RAD001 in patients with advanced NSCLC.	NCT00096486	[107]
Neratinib with and without temsirolimus for patients with HER2-activating mutations in NSCLC.	Phase II (Completed)	HER2-mutant NSCLC	Neratinib, temsirolimus	ORR	NCT01827267	[108]
Trial of continuous once-daily oral treatment using BIBW 2992 (afatinib) plus sirolimus (RapamuneÂ^®^) in patients with NSCLC harboring an EGFR mutation and/or disease progression following prior erlotinib or gefitinib.	Phase I (Completed)	NSCLC	BIBW 2992, sirolimus (rapamycin)	Occurrence of DLT, number of participants with DLT.	NCT00993499	[109]
A study of PDR001 in combination with LCL161, everolimus, or panobinostat.	Phase I (Completed)	Colorectal cancer, non-small cell lung carcinoma (adenocarcinoma), triple-negative breast cancer, renal cell carcinoma	PDR001, LCL161, everolimus, panobinostat, QBM076, HDM201	Incidence of DLTs, frequency of dose interruptions and reductions, frequency and severity of treatment-emergent AEs and SAEs.	NCT02890069	[110]
Phase 1b/2 study of retaspimycin HCl (IPI-504) in combination with everolimus in KRAS-mutant NSCLC.	Phase I, Phase II (Completed)	NSCLC	IPI-504, everolimus	ORR	NCT01427946	[111]
Sirolimus and auranofin in treating patients with advanced or recurrent NSCLC or small cell lung cancer.	Phase I, Phase II (Completed)	Extensive stage small cell lung carcinoma, lung adenocarcinoma, recurrent NSCLC, recurrent small cell lung carcinoma, squamous cell lung carcinoma, Stage IIIA NSCLC, Stage IIIB NSCLC, Stage IV NSCLC	Auranofin, sirolimus	MTD of auranofin, the number and severity of all AEs and PFS.	NCT01737502	[112]
Temsirolimus and radiation for NSCLC.	Phase I (Completed)	NSCLC	Temsirolimus, radiation therapy	Determine the MTD of temsirolimus given with radiation.	NCT00796796	[113]
A Trial of AMG 479, everolimus (RAD001), and panitumumab in patients with advanced cancer—QUILT-3.007.	Phase I (Completed)	Advanced solid tumors, NSCLC	AMG 479, everolimus, panitumumab	To define the MTD and/or recommended RP2TD for the doublet AMG 479 in combination with everolimus in subjects with advanced solid tumors, to define the MTD and/or recommended RP2TD for the triplet AMG 479 in combination with everolimus and panitumumab in subjects with advanced solid tumors.	NCT01061788	[114]
CCI-779 in treating patients with Stage IIIB (with pleural effusion) or stage IV NSCLC.	Phase II (Completed)	Unspecified adult solid tumor, NSCLC	Combination of sorafenib and everolimus	To confirm tumor response.	NCT00079235	[115]
Trial of RAD001 in patients with operable NSCLC.	Phase I (Completed)	Lung cancer	RAD001	Clinical response, effects of RAD001 on the regulation of key proteins involved with the mTOR axis in tumor specimens and buccal mucosa in patients with operable NSCLC, inhibition of proliferation (Ki67) and induction of apoptosis (TUNEL assay) in tumor specimens and buccal mucosa.	NCT00401778	[116]
Safety of everolimus and pemetrexed in lung cancer patients.	Phase I (Completed)	NSCLC	Everolimus, pemetrexed	Establish feasible dose levels/regimens of everolimus combined with pemetrexed chemotherapy.	NCT00434174	[117]
Study investigating the effect of everolimus monotherapy in patients with advanced NSCLC.	Phase II (Completed)	NSCLC	RAD001	Clinical efficacy based on the evaluation of objective tumor response rate.	NCT00124280	[118]
First in human Phase 1 study of AG01 anti-progranulin/GP88 antibody in advanced solid tumor malignancies.	Phase I (Ongoing)	Triple-negative breast cancer, hormone-resistant breast cancer, NSCLC, mesothelioma	AG-01 compound	MTD and/or maximum administered dose (MAD).	NCT05627960	[119]

## Data Availability

The data presented in this study are available in this article.

## Abbreviations

PTEN	Phosphatase and tensin homolog
NSCLC	Non-small cell lung cancer
SCLC	Small cell lung cancer
COPD	Chronic obstructive pulmonary disease
TKI	Tyrosine kinase inhibitors
EGF	Epidermal growth factor
EGFR	Epidermal growth factor receptor
PI3K	Phosphatidylinositol 3-kinase
PIP3	Phosphatidylinositol 3,4,5 trisphosphate
PIP2	Phosphatidylinositol 4,5 bisphosphate
DUB	Deubiquitylates
HDACi	Histone deacetylase inhibitors
5-AZA	5-aza-2′deoxycytidine
HDAC	Histone deacetyl transferase
TSA	Trichostatin A
TGFβRII	Transforming growth factor β receptor type II
TIRF	Total internal reflection fluorescence
MTD	Maximum tolerated dose
DLT	Dose limiting toxicity
AE	Adverse events
TEAEs	Treatment-emergent adverse events
RP2D	Recommended Phase 2 dose
ORR	Overall response rate
TEAE	Treatment-emergent adverse event
SAE	Severe adverse events
Cmax	Maximum observed plasma concentration
Tmax	Time to maximum concentration
